# In silico screening of phytochemicals against chromatin modifier, SETD7 for remodeling of the immunosuppressive tumor microenvironment in renal cancer

**DOI:** 10.1007/s11030-024-11038-w

**Published:** 2024-11-27

**Authors:** Nikhil Gadewal, Diya Patidar, Abhiram Natu, Sanjay Gupta, Virupaksha Bastikar

**Affiliations:** 1 Tata Memorial Centre, Advanced Centre for Treatment, Research and Education in Cancer, Cancer Research Institute, Kharghar, Navi Mumbai, MH 410210 India; 2 Training School Complex, Homi Bhabha National Institute, Anushakti Nagar, Mumbai, MH 400094 India; 3 Center for Computational Biology & Translational Research, Amity Institute of Biotechnology, Amity University, Mumbai, MH India

**Keywords:** SETD7, Phytochemical, Epigenetic, Natural inhibitor

## Abstract

The tumor microenvironment and immune evasion function in a complex cellular network profoundly challenge the clinical outcome of promising therapies. Our recently published study reported that the subset of genes upregulated in ccRCC due to H3K4me1 and DNA demethylation potentially leads to an immunosuppressive environment. Thus, modulating H3K4me1 chromatin modifier SETD7 with a natural inhibitor in combination with immunotherapy might improve the immune landscape for a better therapeutic outcome. The present study was conducted via virtual screening and MD simulation using compounds from the literature, IMPPAT, and SuperNatural database. The phytochemical IMPHY002979 showed better binding affinity and lower energy than the reported R-PFI-2 and cyproheptadine inhibitors. The phytochemicals interact with the SET domain through H-bonding, as confirmed by MD simulation and molecular interaction analysis. Further, the compound was assessed using ADME parameters and free energy estimation, showing better pharmacokinetic properties. Therefore, the non-accessibility of the histone methyltransferase activity domain of SET7 with IMPHY002979 can downregulate H3K4me1 and, thereby, the expression of genes potentially responsible for immunosuppressive TME. Thus, patient stratification based on molecular markers for immunotherapy and combining epigenetic modulators with therapeutic drugs will improve the efficacy of immunotherapy in ccRCC.

**Supplementary Information:**

The online version contains supplementary material available at 10.1007/s11030-024-11038-w.

## Introduction

Renal cancer is a heterogeneous disease with distinct subtypes and specific molecular signatures. The most frequent subtype in renal cancer is the clear cell RCC with overactivation of a specific immune signature, and therefore, treatment approaches should be subtype-specific. Another challenge relates to its chemotherapy and radioresistance patients developing against targeted therapies against VEGFR and the mTOR pathway, so novel therapies are needed. The usual response rate to immunotherapy drugs in cancer patients is between 20 and 50%. Despite the relevance of genomic alterations and instability, the data suggest that the immunogenicity in ccRCC is difficult to explain by the mutational load; therefore, targeting epigenetic modifications may help increase the effectiveness of immune-targeted therapies [1]. Epigenetic modifications, histone, and DNA methylation marks affect immune cells in the tumor microenvironment, provoking an immunosuppressive function and contributing to cancer development [2]. Also, several promoter-methylated genes involved in the various hallmarks of cancer are in RCC, including metabolism, apoptosis, angiogenesis, and cell cycle. Li et al. have shown that HDAC inhibitor combined with IL-2 results in the enrichment of CD25 and CD4-positive T cells and decrease of FoxP3 + ve Tregs, and impeded the development of lung metastases in the mouse model, extending animal survival [3]. The pre-clinical findings led to phase 1/2 trial studies with metastatic forty-seven ccRCC patients, and data showed a positive response with reduced Tregs with entinostat and IL-2 (objective response of 37%), showing the promises for therapeutics [4]. Further, Orillion A and Hashimoto A et al. have also shown that entinostat increases the antitumor effect of immune checkpoint inhibitors, anti-PD-1, in both renal and lung cell carcinoma. The drugs, in combination, improved survival in vivo, changing the immunosuppressive into a tumor-suppressive microenvironment through the downregulation of myeloid-derived suppressor cells and polymorphonuclear neutrophils. These results support combining these epigenetic drugs with immune checkpoint inhibitors [5].

Earlier studies have shown that epigenetic changes in the myeloid-derived suppressor cells (MDSCs), dendritic cells (DCs), tumor-infiltrating lymphocytes (TILs), tumor-associated macrophages (TAMs), natural killer cells (NK cells) and regulatory T cells (Tregs) in the TME persuaded immunosuppressive function through the secretion of inhibitory cytokines, including IL-10 and TGF-β [6]. DNA methylation affects MHCI and II expression, interferes with antigen presentation, and gene-specific methylation of apoptotic proteins inhibits apoptosis, thus impeding the immune system’s recognition [7]. Our earlier study has shown the overexpression of ADA, MSC, RUNX1, TGFA, TREML1, and VWF in the presence of H3K4me1 and H3K4me3 active histone marks and DNA methylation status in the regulatory and gene-body regions of these genes in clear cell renal cell carcinoma (ccRCC) [8]. The overexpression of these genes is associated with myeloid-derived immune suppressor cells, TREM superfamily receptors on mononuclear phagocytes, and the suppression of TH2 gene expression during iTreg differentiation. These upregulated genes lead to an immunosuppressive environment favoring tumor immune escape and may reduce the efficacy of immune checkpoint inhibitors in some ccRCC patients [9].

The epigenetic-induced immunosuppressive function due to the increased level of a subset of genes is partly through H3K4me1. The SET domain containing lysine methyltransferase 7 is the mono-methyltransferase of lysine 4 on histone H3 [10]. SET domain-containing protein has been linked directly to multiple cancer types -breast, liver, stomach, colorectal, lung, and prostate. The SET domain surface HMTase consists of approximately 130 amino acids and exhibits two distinct sites on its surface; one site is for the binding of the methyl-donating cofactor, while the other is for the methyl-accepting substrate. The substrate binding pocket is situated within a deep cavity and is structurally druggable, serving as a common feature across various HMTases. Interestingly, this pocket shares similarities with the ATP-binding pocket found in kinases [11]. SET-domain proteins family consists of seven members SUV39, SET1, SET2, EZ, RIZ, SMYD, and SUV4-20 and orphan members SET7/9 and SET8. The SETD7 has synonyms SET7 and SET9 [12].

Due to the aberrant expression of SETD7 in tumor cells and its high correlation with a bad prognosis for patients with tumors, new inhibitors of SETD7 have been developed as possible therapeutic medicines. R-PFI-2 was identified in 2014 as a substrate-competitive inhibitor of SET7 [13]. It can attach to the SET7 groove and directly contact adenosylmethionine via the catalytic lysine-binding channel. Moreover, the antiallergy medication cyproheptadine is also a SET7 inhibitor, which can lower ERα expression in vivo [14]. The supplementary Table 1, listed the previous studies of SETD7 inhibitors used to build the literature ligand library for virtual screening.

Recently, the group has reported the importance of H3K4me1-mediated upregulation of genes potentially associated with the immunosuppressive environment in ccRCC. Therefore, the present study proposed to identify the inhibitor for SETD7, an enzyme for H3K4me1. The virtual screening, pharmacokinetic properties, and MD simulation studies have shown that the phytochemical IMPHY002979 could be better than reported R-PFI-2, and cyproheptadine inhibitors. Therefore, a natural inhibitor might help modulate the immune landscape for better treatment efficacy.

## Methods

### Datasets and tools

The library of compounds used for virtual screening was obtained from three sources viz 'Literature ligands' contains the literature-reported known compounds, 'SuperNatural' contains anticancer drugs, and 'IMPPAT' contains phytochemical compounds. The conformers were generated using Schrodinger's LigPrep tool. The virtual screening was performed using HTVS (High Throughput Virtual Screening), SP (Standard Precision), and XP (Xtra Precision) docking modes from Schrodinger's Glide tool.

### Library preparation

The construction of the ligand library for SETD7 involved the comprehensive search of research articles and the known inhibitors reported in the public domain databases. The library was compiled by three database sources, viz; literature ligands, SuperNatural database, and IMPPAT database. The literature ligands source consists of 585 inhibitors reported in the published research articles and databases are labeled with alphanumeric numbers (Supplementary Table 1). The inhibitors were coded from research articles as LDCO [15], BPS [16], RPFIANA [17], CYPRO [14], CTFO [18], DSCO [19] and from databases BDDB [20], DGBK [21], CHEMBL [22]. The chemical scaffolds of these literature ligands were captured using the OSRA image reader tool to convert the screen image capture into SMILES strings format and were drawn using Marvin Sketch [23]. The schema of virtual screening of compounds for SETD7 is represented in Fig. 2. The LigPrep tool prepares high-quality small molecule ligand conformers by generating ionization states (pH: 7 ± 2) and stereoisomers with a maximum of thirty-two conformers per ligand [24]. The two-dimensional 585 inhibitors were converted to 1026 three-dimensional conformers using LigPrep. A collection of natural product-based derivatives of over 1441 anticancer drugs from the SuperNatural 3.0 database was processed using the Ligprep tool to obtain 3541 conformers as the natural compound library. The phytochemical compound library of 17,967 compounds was extracted using Python script from the IMPPAT 2.0 database [25]. These two-dimensional SDF compounds were converted to 28,522 three-dimensional conformers by the Ligprep tool. By combining all the compounds from the three libraries, the total compounds for virtual screening were 19,993, with a possible 33,089 conformers.

### Virtual screening

The co-crystallized PDB complexes (PDB ID 4JLG and 5AYF) of SETD7 with R-PFI-2 [13] and cyproheptadine [14] are reported. The 3D structure of SETD7 was used as a receptor from PDB ID 4JLG for virtual screening as PDB ID 5AYF has many missing residues. The residues (266, 267, 335, 336, and 340) that interacted with R-PFI-2 were selected for the grid generation, considering the native ligand coordinates. The Glide docking performs automated orientations of the ligands within the defined grid box to enable rapid evaluation of binding affinities and the identification of potential lead compounds. The docking of known ligand R-PFI-2 with SETD7 was validated by superimposition of the docked complex with the co-crystalized structure of R-PFI-2 with SETD7 (PDB ID 4JLG). The virtual screening was performed using the HTVS, SP, and XP docking modes of Glide, as per the virtual screening schema described in Fig. 2. For the compounds from the IMPPAT database, the conformers were first docked with SETD7 in HTVS mode, followed by SP and later XP docking mode to obtain the best-docked complexes. The conformers of the compounds from the literature ligand and SuperNatural database were docked with SETD7 directly by XP mode to obtain the best-docked complexes. A similar protocol was followed to identify the best-docked conformer for the reference inhibitor R-PFI-2 and cyproheptadine. The shortlisted best-docked complexes from the library of compounds (Fig. 3) and known inhibitors were accessed for the pharmacokinetic properties by predicting molecular descriptors using the QikProp tool from Schrodinger [26].

### Molecular dynamics simulations

The molecular dynamics simulations of eleven docked complexes were performed by the Desmond V5.9 package [27]. The R-PFI-2 and cyproheptadine served as reference compounds for comparative analysis with the nine top-scored compounds. All eleven complexes' systems were built by explicitly adding modified TIP3P water molecules [28]. The OPLS_2005 force-field parameters were used in all the simulations [29]. The docked complexes were simulated for the production run of 100 ns with the recording interval of 100 ps. Before the production run, the systems were relaxed using NVT and NPT ensembles. The trajectories of 1000 frames obtained from the MD simulation were analyzed to gain insights into the protein–ligand complex's stability, conformational changes, and interactions.

### Analysis of the trajectories

The trajectories of the eleven docked complexes were analyzed using the 'Simulation interaction diagram' tool of the Desmond package. Supplementary Fig. 1 depicts the RMSD (Root Mean Square Deviation) plots of SETD7 with the docked compounds. A schematic detailed contacts plots of SETD7 with the compounds highlighting the intermolecular interactions that occur more than 30% of simulation time in Supplementary Fig. 2. The Root Mean Square Fluctuations (RMSF) of the compounds are shown in Fig. 5, along with the intermolecular interactions graph of the residues of SETD7 involved in the different types of interactions with the compounds. To estimate the relative binding affinity of the compounds with SETD7, the MM-GBSA method was implemented using molecular mechanics calculations and solvation models to calculate the free energies of ligand-bound complexes (Delta G bind). The snap-shot of trajectories consists of 100 evenly spaced out representative structures generated for each complex, and MM-GBSA was calculated using the Python script thermal_mmgbsa.py through the Schrodinger power shell [30, 31].

## Results

### Virtual screening of library compounds to identify top-scoring compounds

**Fig. 1:**
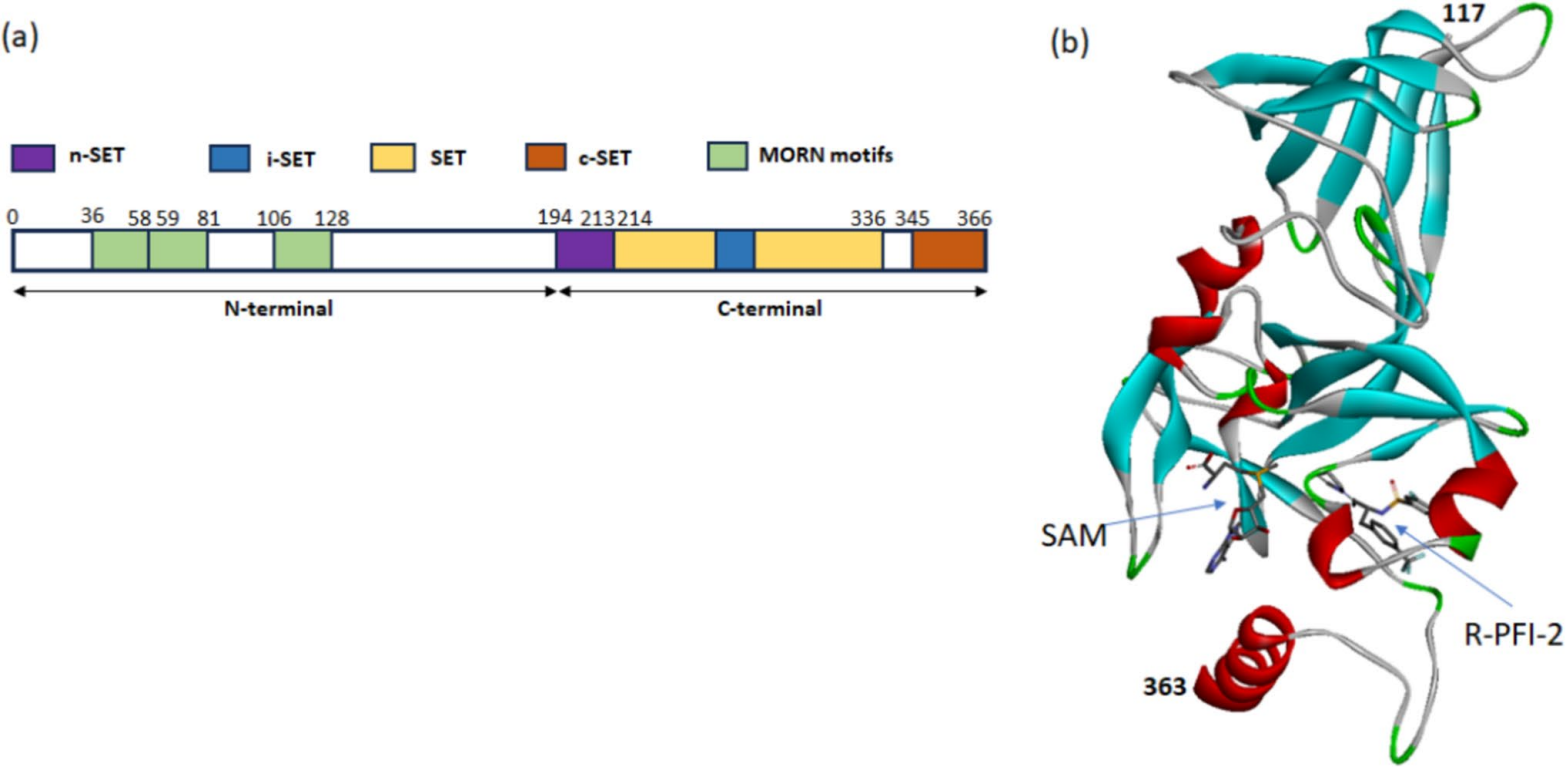
Domains and structure of SETD7: **a** The complete protein domains of SETD7. **b** The crystallized structure of the C-terminal (amino acid 117–363) of SETD7 with inhibitor R-PFI-2 and cofactor SAM (PDB ID: 4JLG)

**Fig. 2:**
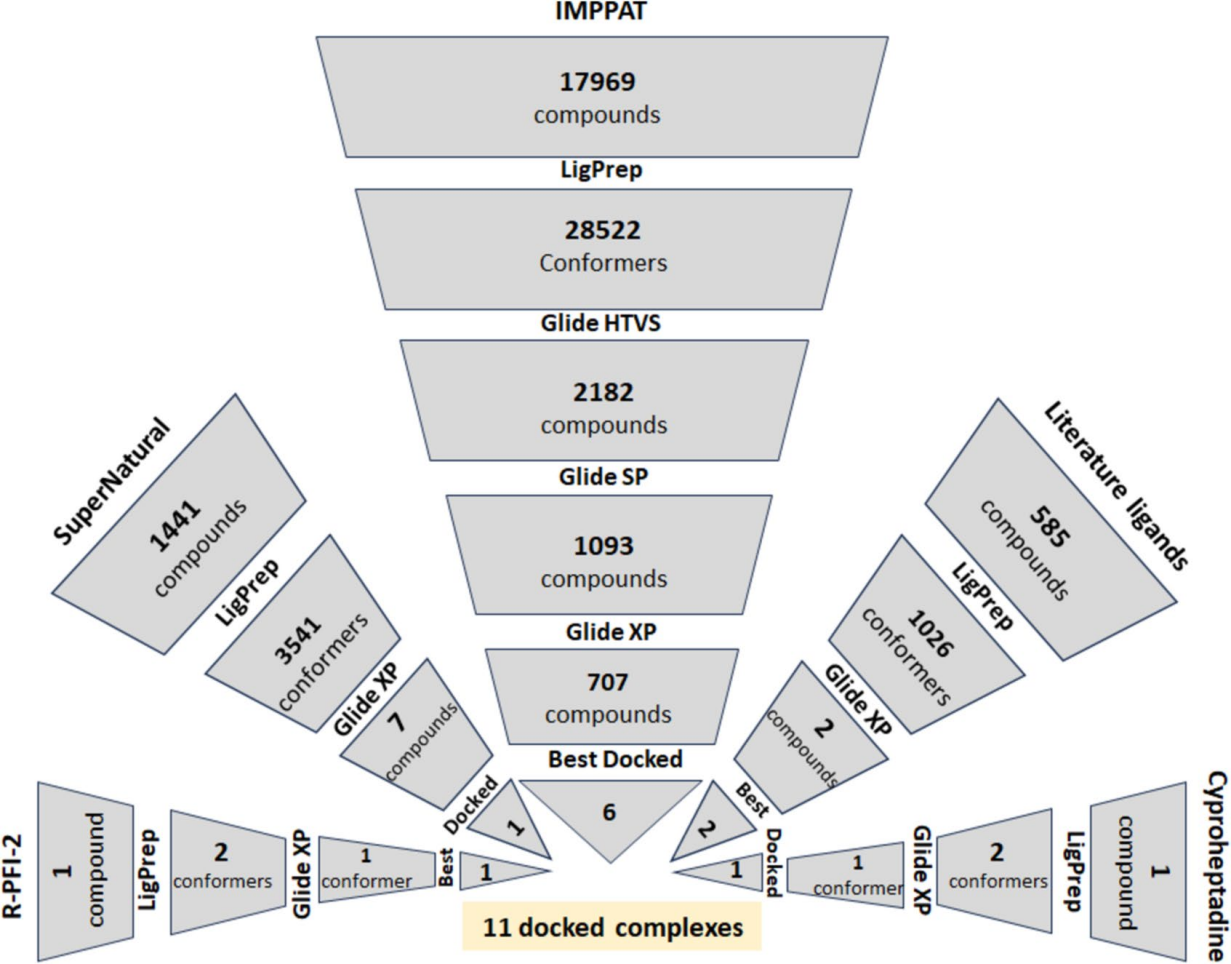
The schema of virtual screening of compounds for SETD7: The virtual screening of compounds from IMPPAT, SuperNatural databases, Literature ligands along with R-PFI-2 and cyproheptadine to obtain top scoring 11 docked complexes. (HTVS: High Throughput Virtual Screening, SP: Standard Precision, XP: Extra Precision)

**Fig. 3:**
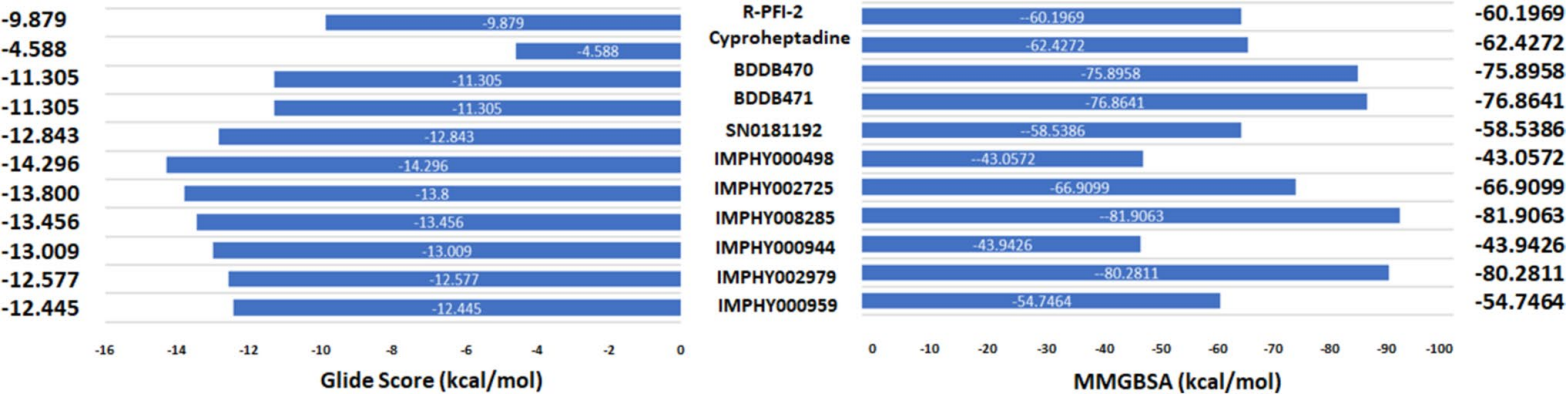
Glide scores and binding free energy of co-crystallized and top hits compounds: The co-crystallized compounds (R-PFI-2 and cyproheptadine) are the known inhibitors and top hits compounds (BDD470, BDD471, SNO181192, IMPHY000498, IMPHY002725, IMPHY008285, IMPHY000944, IMPHY002979, IMPHY000959) with Glide score from docking and MMGBSA (kcal/mol) from MD simulation studies

Before performing virtual screening, the accuracy of docking by Glide was assessed using the RMSD value. The superimposition of a co-crystallized complex of SETD7 with R-PFI-2 (PDB ID 4JLG) and a docked complex of SETD7 R-PFI-2 yielded an RMSD of 0.4751A^o^. This RMSD value indicates that the docking is accurate by the Glide tool. The SETD7 consists of multiple domains, of which the c-terminal domain is involved in the transfer of methyl to the lysine residue of the H3 peptide. (Fig. 1a). The co-crystallized complex of SETD7 with R-PFI-2 (PDB ID: 4JLG) consists of residues from 117 to 363 of c-terminal with the inhibitor R-PFI-2 and cofactor SAM (Fig. 1b). The virtual screening of the library compounds to identify best-docked complexes is reported in Fig. 2. The compounds from the IMPPAT database generated 28,522 conformers for 17,969 compounds. In the first step of HTVS mode, 8125 conformers docked to SETD7 out of 28,522 conformers. In the second step of standard precision (GlideSP) docking, 2182 conformers were selected from 8152, having the glide score cut off at − 6 kcal/mol. Out of 2182 conformers, 1093 conformers docked to SETD7. In the last third step of extra precision docking (GlideXP), 707 conformers were selected from 1093 with the glide score cut off − 6 kcal/mol. Notably, this screening process whittled down the pool to 2182 compounds post-HTVS, reduced to 1093 by GlideSP, and finally to 707 using GlideXP again. Out of 707 Compounds, the top-scoring six compounds of the IMPPAT database were selected, ranging from − 14.295 kcal/mol to − 12.445 kcal/mol glide score. After top-scoring six compounds, the next twelve compounds ranged from − 11.896 kcal/mol to − 11.016 kcal/mol glide score. Therefore, the cut-off of − 12 kcal/mol was selected as the seventh compound's binding energy was 0.545 kcal/mol less than the sixth compound, and the next twelve compounds were within the range of 0.853 kcal/mol. The IMPPAT database has a maximum of 17,969 compounds; the cut-off of − 12 kcal/mol was selected and applied to Supernatural and literature ligand database compounds. Conversely, the Supernatural 3.0 library yielded only a solitary compound meeting the − 12 kcal/mol criteria. Considering the literature ligands, only two compounds were close to − 12 kcal/mol values, which were selected to proceed to the subsequent analysis stage. Nine compounds from three sets of libraries were shortlisted along with R-PFI-2 and cyproheptadine, with Glide scores reported in Fig. 3. The Glide score of R-PFI-2 is − 9.879 kcal/mol, better than the other reference compound, cyproheptadine (− 4.588 kcal/mol). Interestingly, the top-scoring compounds from three sets of libraries have a docking score of more than R-PFI-2 in the range of − 11.305 kcal/mol to − 14.296 kcal/mol. Thus, the virtual screening of the library compounds identified the lead compounds that can bind SETD7 with specific domains with greater binding affinity than R-PFI-2 and cyproheptadine.

### Molecular dynamics simulation and free energy estimation of docked complexes

Molecular dynamics trajectories were explored post the MD simulation to correlate compounds' glide scores and intermolecular interactions with SETD7. The root-mean-square (RMS) deviation plots of SETD7 complexed with R-PFI-2, cyproheptadine, and top hits (nine) compounds for 100 ns were reported in supplementary Fig. 1. Similarly, the contact plots of R-PFI-2, cyproheptadine, and top hits (nine) compounds with SETD7 that occur more than 30.0% of the simulation time of 100 ns were demonstrated in supplementary Fig. 2. The plots summarize the interaction of the atoms of the compounds primarily through hydrogen bonds and non-bonded interactions such as pi–pi and pi-cations that occur more than 30.0% of the simulation time. Supplementary Table 2 summarizes the total number of hydrogen bonds formed between the compounds and SETD7 during 100 ns simulation time. The common and frequent residues involved in interaction with the compound were Gly336 and Ser268. Prime MMGBSA module of Schrodinger calculates the total energy of the complex along with contributions of different energies. Figure 3 highlights the total energy of the SETD7-compounds complexes, revealing a discernible pattern in the MMGBSA values, ranging from − 42 kcal/mol to − 87 kcal/mol. Interestingly, the cyproheptadine MMGBSA value was comparable to another reference compound, R-PFI-2, even though the docking glide score was nearly half of the R-PFI-2. The compounds from the IMPPAT database scored the highest MMGBSA, corroborating with the Glide docking scores. Based on the Glide docking score and total free energy of the complexes, the compounds IMPHY008285 and IMPHY002979 were selected as the lead compounds as they have the highest MMGBSA values of − 81.9063 kcal/mol and − 80.2811 kcal/mol, which are more than thirty units compared to other compounds from the IMPPAT database.

**Fig. 4:**
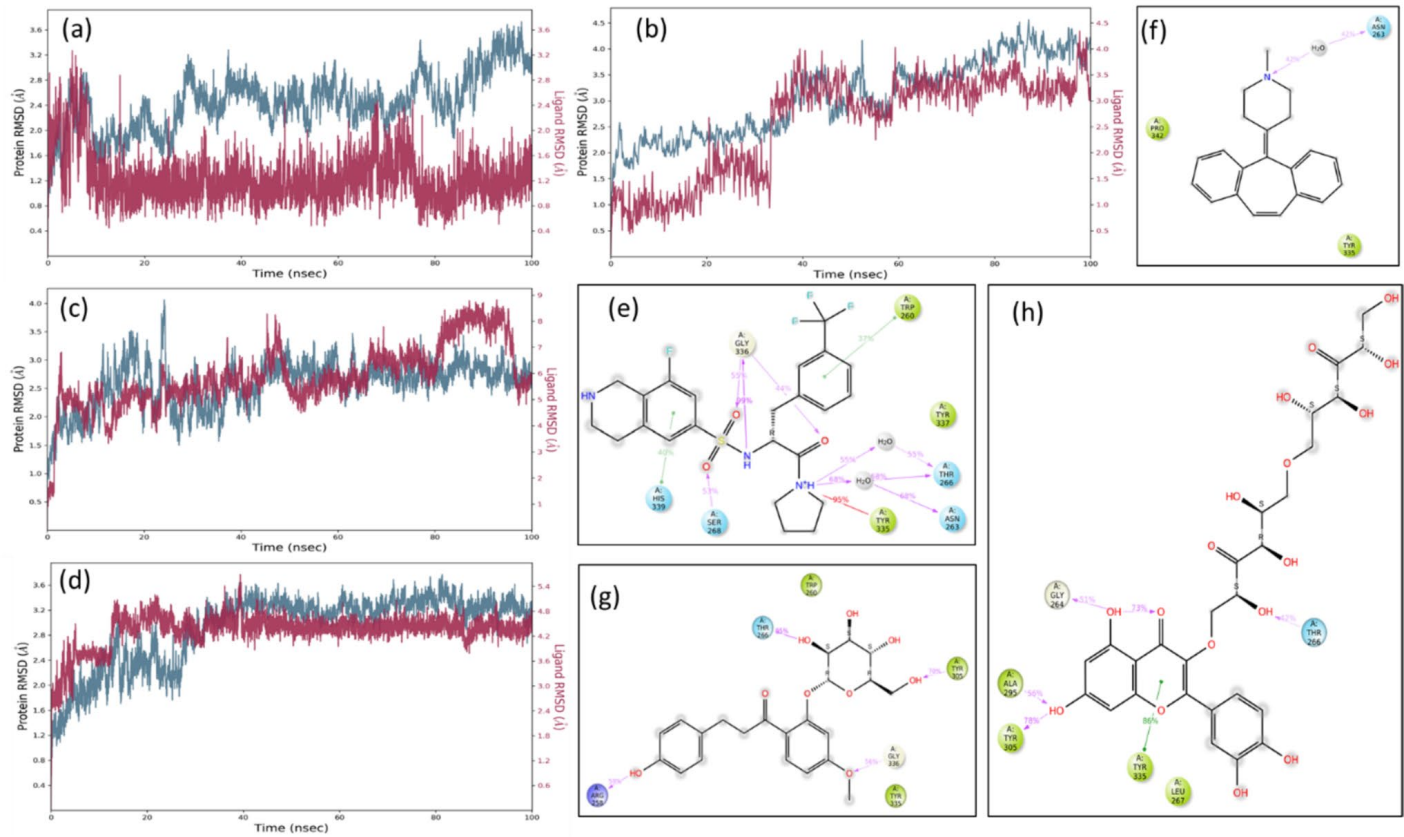
Complex RMSD plots of SETD7 (cyan) and compounds (red) from MD simulation study for 100 ns **a** R-PFI-2, **b** cyproheptadine **c** IMPHY008285 and **d** IMPHY002979. Intermolecular contacts of compound-SETD7 that occur more than 30.0% of the simulation time of 100 ns **e** R-PFI-2, **f** cyproheptadine **g** IMPHY008285 and **h** IMPHY002979. The interactions in pink (H-bond), green (pi-pi), and red (pi-cation) with residues of SETD7 (polar in blue color, hydrophobic in green color)

**Fig. 5:**
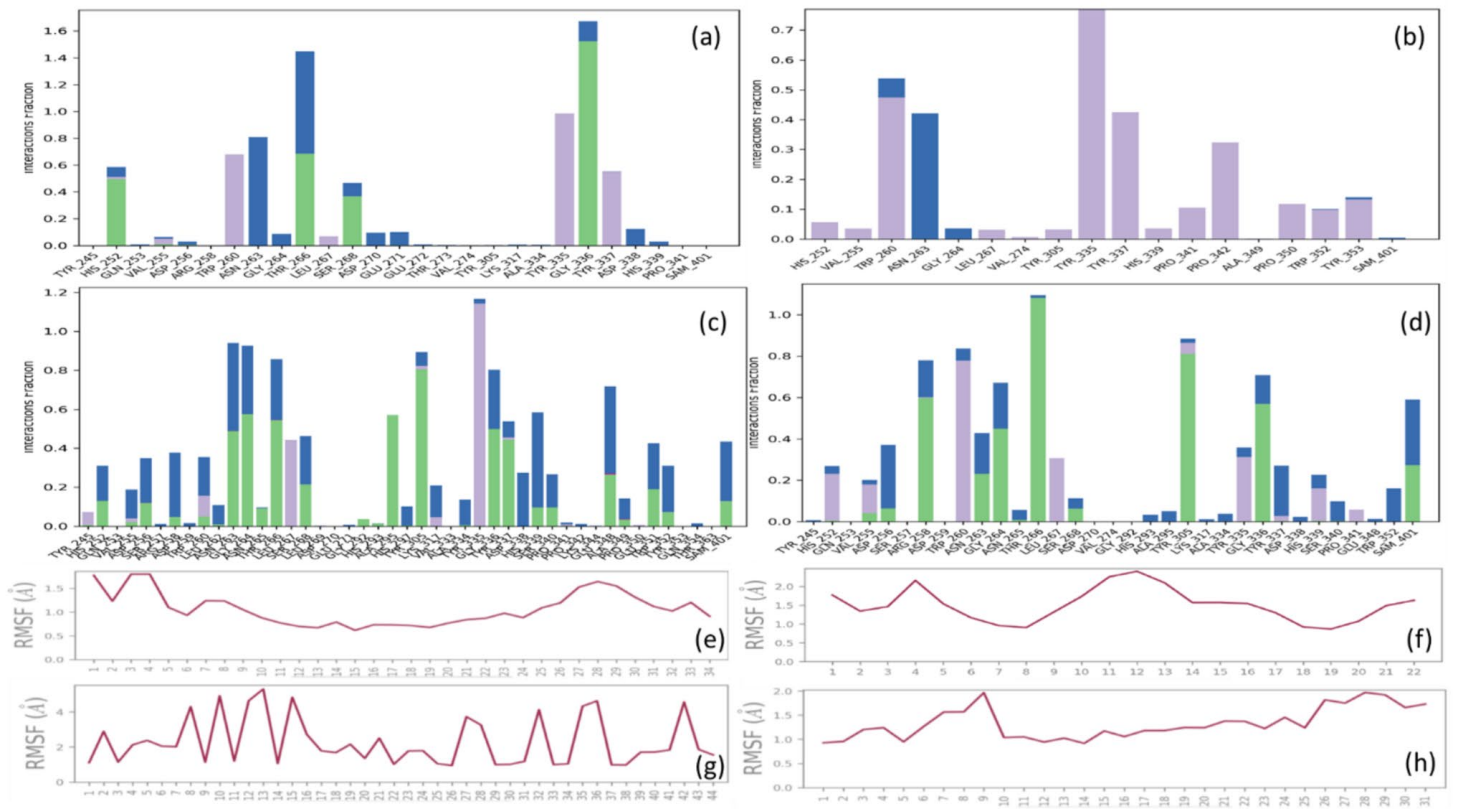
Total known intermolecular interactions graph of compound-SETD7 that occur during the simulation time of 100 ns **a** R-PFI-2, **b** cyproheptadine **c** IMPHY008285 and **c** IMPHY002979. The H-bond interactions in green, hydrophobic in purple, and water bridges in blue. The RMSF plots of compounds **d** R-PFI-2, **e** IMPHY008285, and **f** IMPHY002979 for 100 ns

To further investigate the intermolecular interactions between the known R-PFI-2 and cyproheptadine along with two lead compounds, IMPHY008285 and IMPHY002979, the comparative analysis of RMSD and contact plots are summarized in Fig. 4. The RMSD of SETD7 of all four complexes and RMSD of R-PFI-2, cyproheptadine, and IMPHY002979 were in the acceptable range of 1–3 A. However, the RMSD of IMPHY008285 deviated more than 6 A. The deviation is from the highly fluctuating RMSF range from 1 to 5 A (Fig. 5g) compared to the 1–2 A RMSF range of R-PFI-2, cyproheptadine and IMPHY002979 (Figs. 5e, f, h). It is interesting to note that IMPHY008285 consists of 44 atoms (Fig. 5g) which form a total of 22 hydrogen bonds with SETD7 (Fig. 5b) throughout the simulation, but only 5 hydrogen bonds [Thr266 (42%), Gly264 (51%), Ala295 (56%), Tyr305 (78%), Tyr335 (86%)] maintained the contacts with the residues more than 30% of simulation time of 100 ns (Fig. 4h). Contrastly, R-PFI-2 consists of 34 atoms (Fig. 5e) which form total four hydrogen bonds with SETD7 (Fig. 5a) throughout the simulation, but only two hydrogen bonds [Gly336 (99%, 55%, 44%) and Ser268 (53%)] maintained the direct contacts and other two [Thr266 (55% and 68%) and Asn263 (68%)] made water-mediated contacts with the residues more than 30% of simulation time of 100 ns (Fig. 4e). His339, Tyr335, and Trp60 also made hydrophobic contacts (Fig. 4e). The other reference compound, cyproheptadine, consists of 22 atoms (Fig. 5f), which forms mostly hydrophobic interactions with SETD7 and only a single water-mediated hydrogen bond with Asn263 (42%) more than 30% of simulation time of 100 ns (Fig. 4f). The compound IMPHY002979 (31 atoms) behaves similarly to R-PFI-2, forming 10 hydrogen bonds with SETD7 (Fig. 5d) throughout the simulation. Still, only 4 hydrogen bonds [Thr266 (95%), Gly336 (56%), Arg258 (59%), Tyr305 (70%)] maintained contact with the residues for more than 30% of the simulation time of 100 ns (Fig. 4g). Based on the RMSD, RMSF, and the contact plots, it is clear that IMPHY002979 showed stable interaction with SETD7 while having similar free energy compared to IMPHY008285. Therefore, IMPHY002979 appears to be the best phytochemical for evaluating pharmacokinetic properties.

### The mechanistic insights of the interaction of SETD7 residues with the inhibitors

**Fig. 6:**
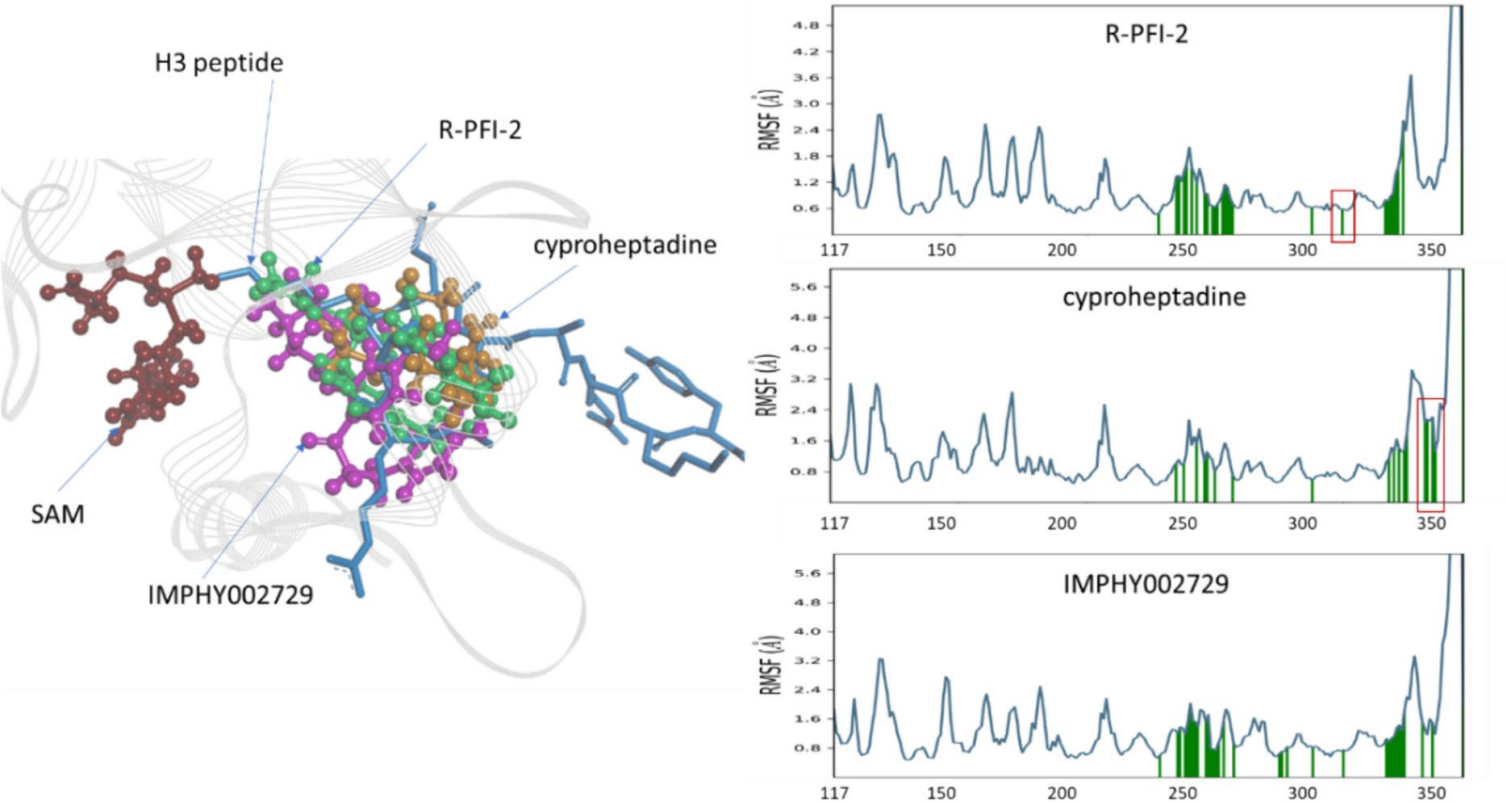
**a** Superimposition of R-PFI-2 (green), cyproheptadine (orange), and IMPHY002979 (pink) with the SETD7-bound conformation of a substrate histone peptide (blue). The RMSF of SETD7 complexed with the inhibitors. **b** RMSF of SETD7 R-PFI-2 complex **c** RMSF of SETD7 cyproheptadine complex **d** RMSF of SETD7 IMPHY002979 complex

The co-crystallized structure of SETD7 with histone H3 peptide and S-adenosyl-l-methionine (PDB ID: 1O9S) demonstrated that the peptide substrate and cofactor bind on opposite surfaces of the enzyme [32]. By introducing its side chain into a small channel that passes through the enzyme and joins the two surfaces, the fourth amino acid of the histone H3 peptide gains access to the active site of SETD7 and the S-adenosyl-l-methionine (Fig. 6a). This co-crystallized complex was superimposed with inhibitors R-PFI-2, cyproheptadine, and IMPHY002979 to confirm that inhibitors are docked at the same position where the lysine residue of the H3 histone interacts (Fig. 6a). The RMSF plots of the SETD7 docked complexes show the residues involved in bonded and non-bonded interactions with R-PFI-2, cyproheptadine, and IMPHY002979 in green color vertical bars (Fig. 6b, c, d). The R-PFI-2 shows the interaction with Tyr317, while cyproheptadine does not interact. Similarly, cyproheptadine interacts with Pro350, Trp352, and Tyr353, and R-PFI-2 does not. But IMPHY002979 shows the interaction with both Tyr317 and Trp352. Interestingly, cyproheptadine forms only hydrophobic interaction, which is reflected in less docking score (− 4.588 kcal/mol) than R-FFI-2 (− 9.879 kcal/mol). However, the binding free energy of both the known inhibitors are similar. The IMPHY002979 has a free energy score of − 80.2811 kcal/mol, which may bind better with SETD7 than known inhibitors. Thus, IMPHY002979 can modulate the activity of SETD7 by restricting the transfer of methyl group to the lysine residue of histone H3 lysine better than known inhibitors.

### Comparison of pharmacokinetic properties of IMPHY002979 with known inhibitors

The molecular descriptors were predicted using the QikProp tool from Schrodinger to investigate the drug-like properties of these lead compounds. Out of the 50 calculated descriptors, Mol_mw, CNS, SASA, donorHB accptHB, QPlogHERG, QPPCaco, QPPMDCK, QPlogKp, Oral absorption and rule of five were selected to access its drug-like properties (Table 1). IMPPAT inhibitors, IMPHY002979, fall within the moderate molecular weight range of 130.0 to 725.0 and have lower molecular weight than R-PFI-2. The central nervous system activity of IMPHY002979 is − 2, which indicates an inactive state, suggesting limited neurological impact. However, the CNS activity of R-PFI-2 is 1, which may cause severe interactions of neurotransmitter systems. HERG K + channel blockage, reflected in QPlogHERG values, is marginal below − 5, highlighting concern for IMPHY002979, which differs marginally from R-PFI-2 and cyproheptadine. The gut-blood barrier (QPPCaco) and blood–brain barrier (QPPMDCK) values are in the poor range, which is not a significant concern for oral usage. This property is overcome by the human oral absorption assessments, in which IMPHY002979 was categorized as medium to low level. Moderate to high skin permeability (QPlogKp − 8.0 to − 1.0) and moderate oral absorption (human oral absorption level 2) align with drug-like characteristics, while adherence to Lipinski's rule of five underscores its compliance. In summary, the IMPHY002979 compound complies with pharmacokinetic benchmarks as a viable candidate for further in-depth wet-laboratory investigations and development.

**Table 1:**
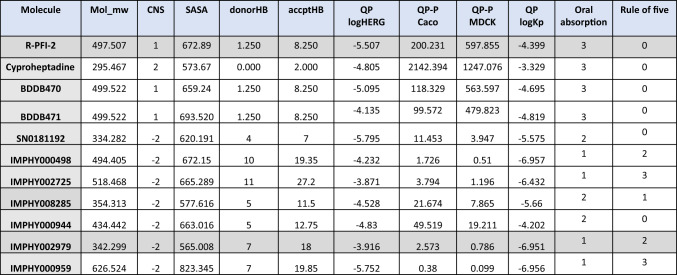
The pharmacokinetic and physicochemical (ADME) properties of R-PFI-2, cyproheptadine, and nine compounds predicted by QikProp

## Discussion

The immunosuppressive environment facilitated or induced by cancers mainly affected the efficacy of immunotherapies, leading to immune escape and tumor progression. An in silico study has shown that H3K4me1-regulated overexpression of a group of genes potentially favors an immunosuppressive microenvironment in clear cell renal cell carcinoma [9]. SET domain containing 7 (SETD7), a member of histone methyltransferases for H3K4me1, is abnormally expressed in multiple tumor types, including clear cell renal cell carcinoma (ccRCC).

The current study focused on the virtual screening of phytochemical compounds for their inhibitory potential against a specific domain of methyltransferase, SETD7. The docking and free energy calculation of the reference inhibitors R-PFI-2 and cyproheptadine have low docking and MMGBSA scores compared to the phytochemical inhibitor IMPHY002979. The trajectory analysis and contact plots also substantiate that phytochemical IMPHY002979 has a stable and better binding affinity than R-PFI-2 and cyproheptadine. Moreover, free binding energy calculations and ADMET investigations unveiled a natural compound, IMPHY002979, with the highest binding energy values compared to the reference molecule. Natural products are significant resources for drug development. The IMPHY002979 is extracted from the medicinal plant *Broussonetia papyrifera*, a deciduous tree that grows naturally in Asia and Pacific Rim countries such as China and Thailand [33]. Since ancient times, its roots, bark, and fruits have been used as traditional Chinese medicine for antioxidant, antibacterial, anti-inflammatory, and antitumor activities [34]. The IMPHY002979 compound belongs to Class Flavonoids, Subclass Chalcones, and dihydrochalcones, and it has a chemical name, 4-O-methyldavidioside [25]. The present analysis showed for the first time that IMPHY002979 is a potent inhibitor against SETD7 with a potential binding pocket. The active ingredient 4'-O-Methyldavidioside in IMPHY002979 compound has cytotoxic activity against MCF-7, NCI-H460, and SF-268 cancer cell lines [35].

In continuation, as per the IMPPAT database, the IMPHY002979 is not an inhibitor of cytochrome enzymes (CYP1A2, CYP2C19, CYP2C9, CYP2D6, CYP3A4), which suggests that the potential of drug-drug interactions would be less (https://cb.imsc.res.in/imppat/admetproperties/IMPHY002979). ADMET properties at IMPPAT also reveal that IMPHY002979 is predicted to be a substrate for p-glycoprotein export transporters, which may result in less oral absorption and drug-drug interactions. The drug-likeliness properties of IMPHY002979 are predicted by the RDKit tool by seven rules (Lipinski's, Ghose, Veber, Egan, GSK, Pfizer, and QEDw) at IMPPAT database (https://cb.imsc.res.in/imppat/druglikeproperties/IMPHY002979). Phytochemical IMPHY002979 passed by Lipinski's, Ghose's, Pfizer's, and QEDw scores; however, since it is a substrate for p-glycoprotein, the Veber rule, the Egan rule, and the GSK 4/400 rule, the IMPHY002979 does not exhibit good oral bioavailability. Therefore, alternate administration or higher doses should be considered if the molecule is effective. Additionally, the pharmacokinetic properties assessed in our study suggest that IMPHY002979 complies with drug-like characteristics and, therefore, is a promising and specific natural compound SETD7 inhibitor.

Several studies have suggested that epigenetic modifier inhibitors display intrinsic immunomodulatory properties. The class 1 HDAC inhibitor entinostat can suppress T regulatory cells and enhance the antitumor effect of PD-1 inhibition, improving immunotherapy response in murine renal cancer models [5]. Additionally, combining valproic acid and IFN-alpha to treat the metastatic RCC cell line Caki-1 can increase the transcription of chemokines and integrins after three to five days of exposure [36]. The combination of panobinostat (HDACi) with bortezomib (a proteasome inhibitor) and dexamethasone (an immunomodulatory drug) improves progression-free survival in patients with MM and is approved by the FDA [37]. Recent studies highlighted the potential for DNA methyltransferase 1 (DNMT1i) and EZH2i inhibitors to rewire a 'cold' to a 'hot' microenvironment [37]. Moreover, HMT inhibitors have been shown to play an important role in reversing Treg cells' immunosuppression with checkpoint blockade. Therefore, checkpoint blockade with EZH2 inhibitors, tazemetostat, and CPI-205 is under clinical trials, and FDA approved [39, 40]. The adjunction of the BETi JQ1 to immune-stimulating therapies (anti-PD-1 or anti-CD137 antibodies) improved Eμ-Myc lymphoma-bearing mice's survival [38]. Another study analyzed changes in mRNA expression in metastatic castration-resistant prostate cancer patients treated with ZEN-3694 [39]. This BETi induced a significant downregulation of several checkpoints receptors (including PD-L1, TIM3, and A2AR) as well as cytokines/chemokines (IL-8, CXCR1, and CCR2), while co-stimulatory factors remained unchanged [37].

Earlier studies have shown that in a sub-group of ccRCC patients, T cells cannot infiltrate the tumor site to produce an immunosuppressive tumor environment [36]. Moreover, SETD7 interacts with p53, ERα, TAF7, and YAP1 and regulates oncogenic pathways such as Wnt/ß-catenin and Hippo-pathway, contributing to numerous biological processes, including tumor immune microenvironment [40]. ß-catenin promotes a non-inflammatory environment within tumors by preventing CD8 + T cell activation and promoting Treg cell infiltration [41]. Due to the disruption of immunological checkpoints in the tumor microenvironment, the alterations result in resistance to ICIs, particularly anti-PD-L1/PD-1 signaling inhibitors, which decrease T cell suppression and boost the antitumor immune response [42]. Moreover, SETD7 can methylate p65, thereby inhibiting the expression of NF-κB and is a positive regulator of Transforming Growth Factor Beta (TGF-ß) production, contributing to tumorigenesis and immune suppression [43]. IMPHY002979, SETD7 inhibitor can potentially enhance the immune response or suppress the immune suppressor. Moreover, a recent database and web platform study investigated immunotherapy response biomarkers in a large cohort of solid tumor samples and predicted SETD7 as one of the druggable gene candidates linked to anti-PD-1 resistance [37, 42].

The work provides a scientific basis for the comprehensive application of a natural compound, IMPHY002979, in potentially regulating the epigenomic landscape and laying the foundation for its follow-up in vitro and in vivo research. Moreover, SETD7 and H3K4me1 may serve as biomarkers for patient stratification, and the application of combination immunotherapy approaches with epigenetic modifiers inhibitors. This may influence metabolic and transcriptional reprogramming in immune cells, resulting in inhibition of immunosuppressive or 'cold' tumor microenvironment and may re-sensitize the sub-group of patients, thus maximizing the chance for therapeutic success.

## Supplementary Information

Below is the link to the electronic supplementary material.

Supplementary file1 (TIF 3311 kb)

Supplementary file2 (TIF 3137 kb)

Supplementary file3 (PPTX 43 kb)

Supplementary file4 (PPTX 44 kb)

## Data Availability

No datasets were generated or analysed during the current study.
