# Supplementary figures and images for: In silico screening of phytochemicals against chromatin modifier, SETD7 for remodeling of the immunosuppressive tumor microenvironment in renal cancer
Source: Mol Divers. 2024 Nov 27;29(5):4359–69. doi: 10.1007/s11030-024-11038-w (PMC12454513; doi:10.1007/s11030-024-11038-w)

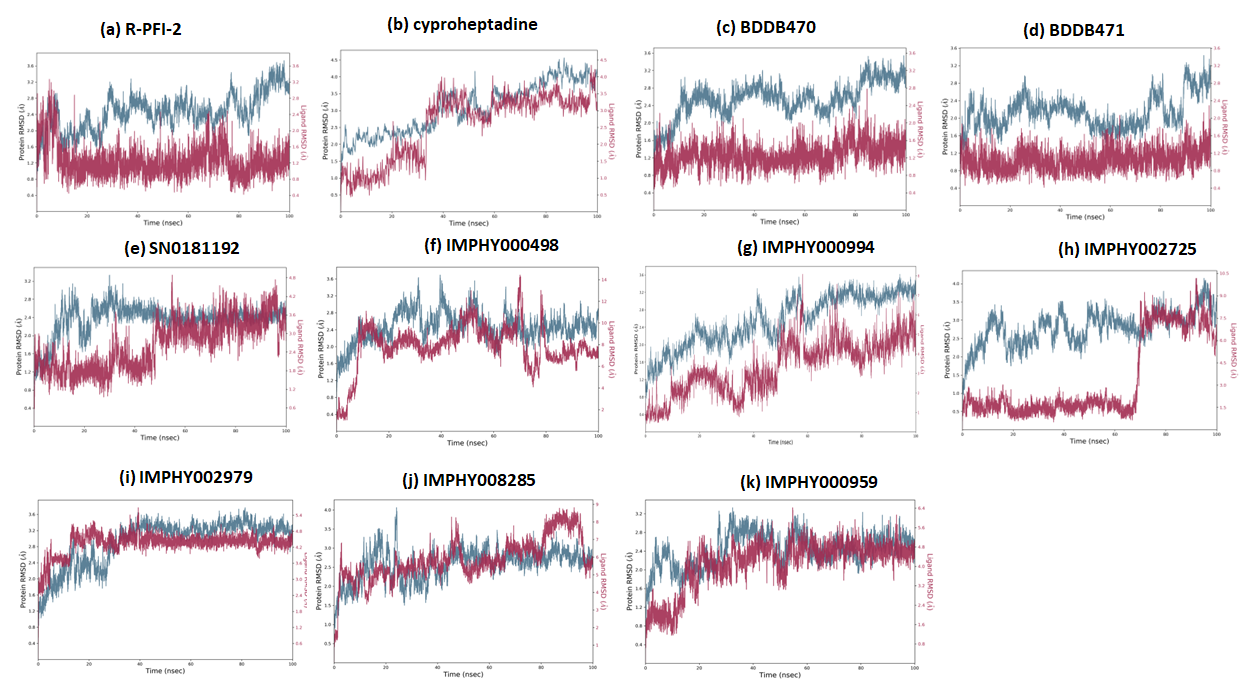

Supplement: Supplementary file 1 — Supplementary file1 (TIF 3311 kb) [file 11030_2024_11038_MOESM1_ESM.tif]

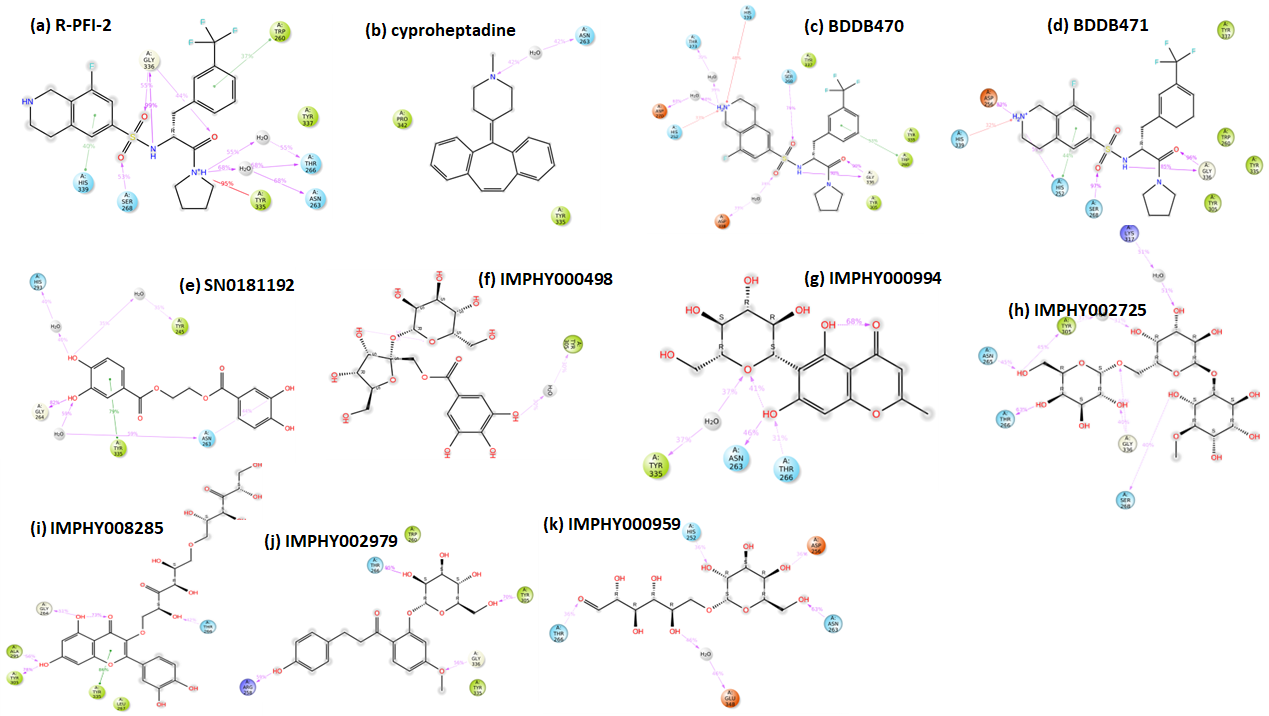

Supplement: Supplementary file 2 — Supplementary file2 (TIF 3137 kb) [file 11030_2024_11038_MOESM2_ESM.tif]
