# Supplementary material for: In silico screening of phytochemicals against chromatin modifier, SETD7 for remodeling of the immunosuppressive tumor microenvironment in renal cancer
Source: Mol Divers. 2024 Nov 27;29(5):4359–69. doi: 10.1007/s11030-024-11038-w (PMC12454513; doi:10.1007/s11030-024-11038-w)
Supplement: Supplementary file 3 — Supplementary file3 (PPTX 43 kb) [file 11030_2024_11038_MOESM3_ESM.pptx]

## Slide 1
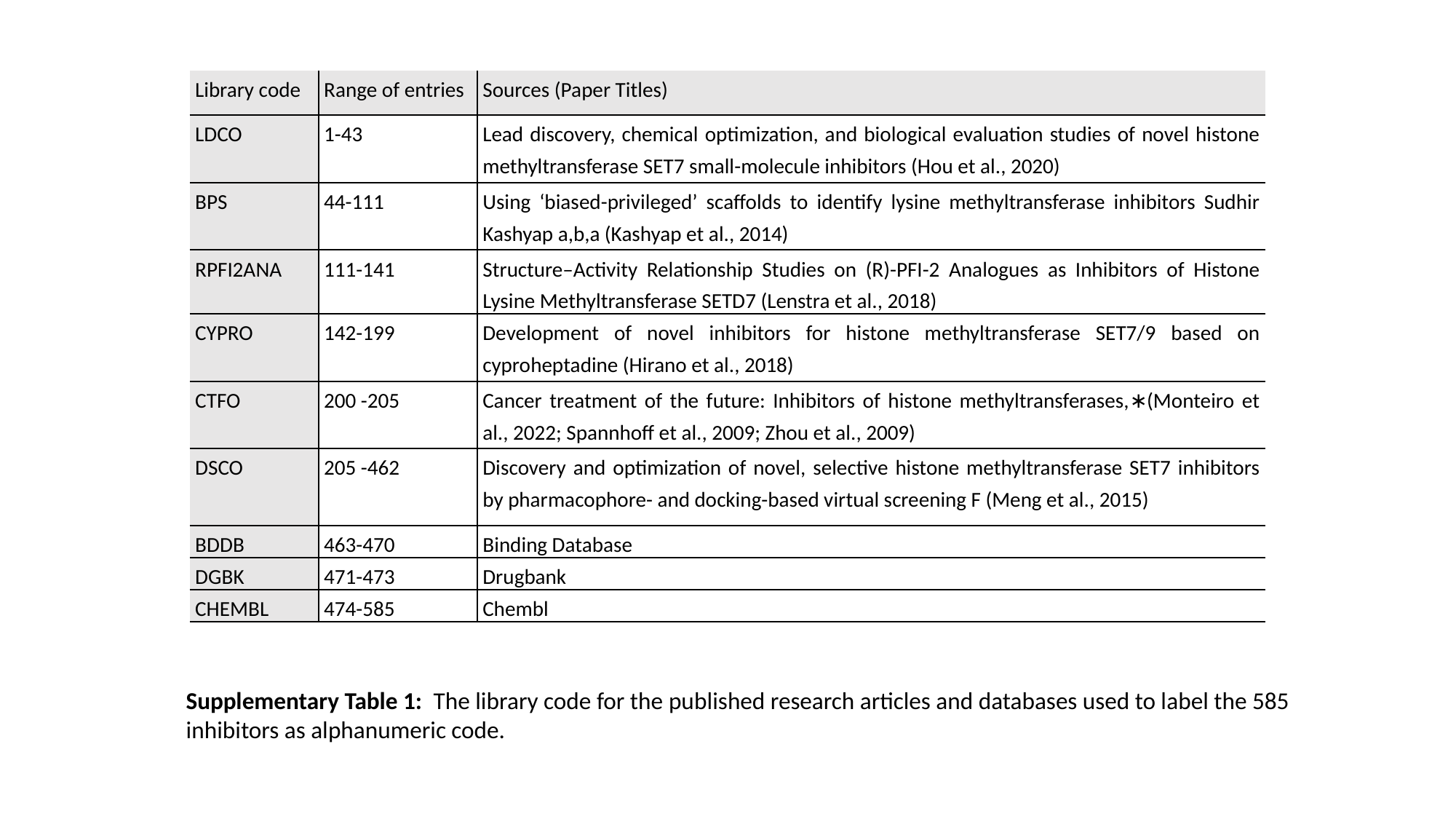

| Library code | Range of entries | Sources (Paper Titles) |
| --- | --- | --- |
| LDCO | 1-43 | Lead discovery, chemical optimization, and biological evaluation studies of novel histone methyltransferase SET7 small-molecule inhibitors (Hou et al., 2020) |
| BPS | 44-111 | Using ‘biased-privileged’ scaffolds to identify lysine methyltransferase inhibitors Sudhir Kashyap a,b,a (Kashyap et al., 2014) |
| RPFI2ANA | 111-141 | Structure–Activity Relationship Studies on (R)-PFI-2 Analogues as Inhibitors of Histone Lysine Methyltransferase SETD7 (Lenstra et al., 2018) |
| CYPRO | 142-199 | Development of novel inhibitors for histone methyltransferase SET7/9 based on cyproheptadine (Hirano et al., 2018) |
| CTFO | 200 -205 | Cancer treatment of the future: Inhibitors of histone methyltransferases,∗(Monteiro et al., 2022; Spannhoff et al., 2009; Zhou et al., 2009) |
| DSCO | 205 -462 | Discovery and optimization of novel, selective histone methyltransferase SET7 inhibitors by pharmacophore- and docking-based virtual screening F (Meng et al., 2015) |
| BDDB | 463-470 | Binding Database |
| DGBK | 471-473 | Drugbank |
| CHEMBL | 474-585 | Chembl |
Supplementary Table 1: The library code for the published research articles and databases used to label the 585 inhibitors as alphanumeric code.
