# Supplementary material for: In silico screening of phytochemicals against chromatin modifier, SETD7 for remodeling of the immunosuppressive tumor microenvironment in renal cancer
Source: Mol Divers. 2024 Nov 27;29(5):4359–69. doi: 10.1007/s11030-024-11038-w (PMC12454513; doi:10.1007/s11030-024-11038-w)
Supplement: Supplementary file 4 — Supplementary file4 (PPTX 44 kb) [file 11030_2024_11038_MOESM4_ESM.pptx]

## Slide 1
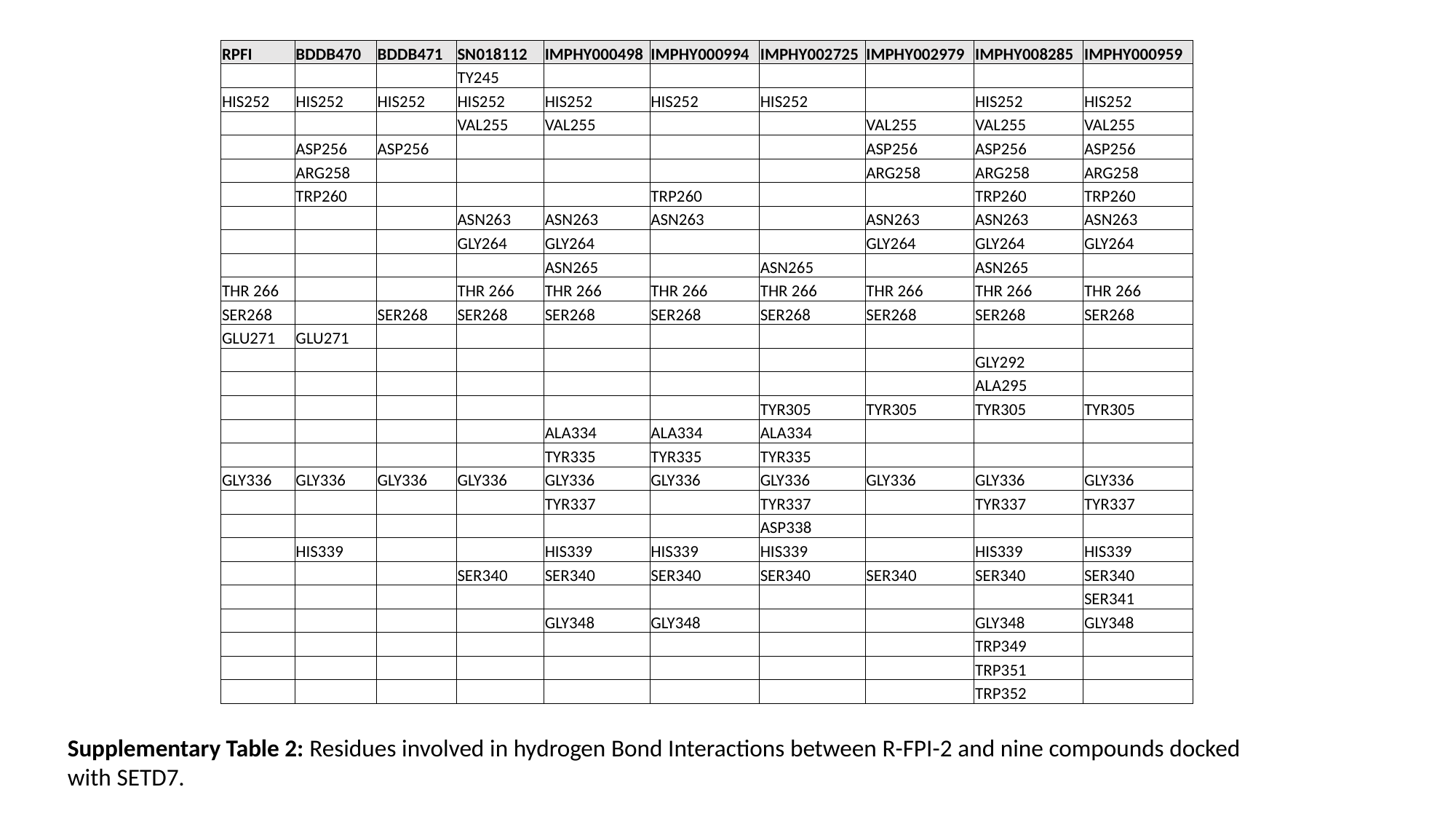

| RPFI | BDDB470 | BDDB471 | SN018112 | IMPHY000498 | IMPHY000994 | IMPHY002725 | IMPHY002979 | IMPHY008285 | IMPHY000959 |
| --- | --- | --- | --- | --- | --- | --- | --- | --- | --- |
| | | | TY245 | | | | | | |
| HIS252 | HIS252 | HIS252 | HIS252 | HIS252 | HIS252 | HIS252 | | HIS252 | HIS252 |
| | | | VAL255 | VAL255 | | | VAL255 | VAL255 | VAL255 |
| | ASP256 | ASP256 | | | | | ASP256 | ASP256 | ASP256 |
| | ARG258 | | | | | | ARG258 | ARG258 | ARG258 |
| | TRP260 | | | | TRP260 | | | TRP260 | TRP260 |
| | | | ASN263 | ASN263 | ASN263 | | ASN263 | ASN263 | ASN263 |
| | | | GLY264 | GLY264 | | | GLY264 | GLY264 | GLY264 |
| | | | | ASN265 | | ASN265 | | ASN265 | |
| THR 266 | | | THR 266 | THR 266 | THR 266 | THR 266 | THR 266 | THR 266 | THR 266 |
| SER268 | | SER268 | SER268 | SER268 | SER268 | SER268 | SER268 | SER268 | SER268 |
| GLU271 | GLU271 | | | | | | | | |
| | | | | | | | | GLY292 | |
| | | | | | | | | ALA295 | |
| | | | | | | TYR305 | TYR305 | TYR305 | TYR305 |
| | | | | ALA334 | ALA334 | ALA334 | | | |
| | | | | TYR335 | TYR335 | TYR335 | | | |
| GLY336 | GLY336 | GLY336 | GLY336 | GLY336 | GLY336 | GLY336 | GLY336 | GLY336 | GLY336 |
| | | | | TYR337 | | TYR337 | | TYR337 | TYR337 |
| | | | | | | ASP338 | | | |
| | HIS339 | | | HIS339 | HIS339 | HIS339 | | HIS339 | HIS339 |
| | | | SER340 | SER340 | SER340 | SER340 | SER340 | SER340 | SER340 |
| | | | | | | | | | SER341 |
| | | | | GLY348 | GLY348 | | | GLY348 | GLY348 |
| | | | | | | | | TRP349 | |
| | | | | | | | | TRP351 | |
| | | | | | | | | TRP352 | |
Supplementary Table 2: Residues involved in hydrogen Bond Interactions between R-FPI-2 and nine compounds docked with SETD7.
